# Corticostriatal pathways contribute to the natural time course of positive mood

**DOI:** 10.1038/ncomms10065

**Published:** 2015-12-07

**Authors:** Roee Admon, Diego A. Pizzagalli

**Affiliations:** 1 McLean Hospital and Harvard Medical School, de Marneffe Building, Room 233A, 115 Mill Street, Belmont, Massachusetts 02478-9106, USA

## Abstract

The natural time course of mood includes both acute responses to stimuli and spontaneous fluctuations. To date, neuroimaging studies have focused on either acute affective responses or spontaneous neural fluctuations at rest but no prior study has concurrently probed both components, or how mood disorders might modulate these processes. Here, using fMRI, we capture the acute affective and neural responses to naturalistic positive mood induction, as well as their spontaneous fluctuations during resting states. In both healthy controls and individuals with a history of depression, our manipulation acutely elevates positive mood and ventral striatum activation. Only controls, however, sustain positive mood over time, and this effect is accompanied by the emergence of a reciprocal relationship between the ventral striatum and medial prefrontal cortex during ensuing rest. Findings suggest that corticostriatal pathways contribute to the natural time course of positive mood fluctuations, while disturbances of those neural interactions may characterize mood disorder.

## Abstract (web summary)

Positive mood can occur as a result of a stimulus or spontaneously. Admon and Pizzagalli measure variations in neural responses to a positive stimulus over time, and identify cortico-striatal interactions associated with sustained positive mood which are reduced in individuals with a history of depression.

## Graphical abstract

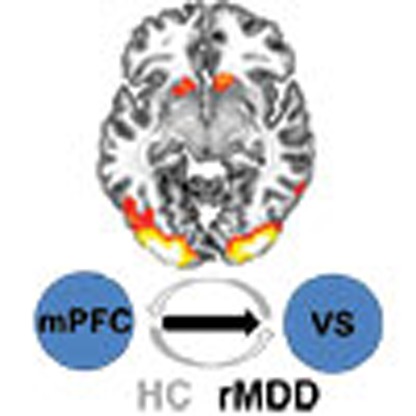

Mood affects how we perceive and respond to daily experiences, yet such experiences often trigger subsequent fluctuations in mood. Over the years, a plethora of experimental manipulations have been used to acutely induce a specific mood state. Interestingly, induction of acute positive affect has been more challenging than of negative mood, particularly if a naturalistic induction is targeted in which participants are unaware of its purpose^1^. Even upon successful induction of acute affective states, extant studies have typically neglected to probe mood fluctuations following stimuli offset, which may have spontaneously occurred subsequent to the acute response. As a result, the natural time course of positive mood fluctuations remains largely unexplored.

Along similar lines, neuroimaging studies, to date, have separately investigated the neural bases of acute responses to positive stimuli or spontaneous functional fluctuations in the absence of external stimuli. In this context, both human and nonhuman studies have established the central role of a corticostriatal neural circuit, particularly the ventral striatum (VS; nucleus accumbens), in mediating acute responses to various positive stimuli^2^,^3^. Conversely, spontaneous neural function (for example, during resting state) reliably engages the default mode network (DMN)—a network of regions encompassing the medial prefrontal, medial temporal and posterior cingulate cortices, which are hypothesized to support distinct aspects of internally oriented and self-referential thoughts^4^,^5^,^6^. Given that the natural time course of positive mood involves both acute responses to external positive stimuli and spontaneous fluctuations, examining the relation between VS and DMN may help to advance our understanding of the neural substrates of positive mood. Specifically, it is currently largely unknown whether (1) acute VS responses to positive stimuli interact with the DMN during ensuing rest, and (2) such interactions contribute to the natural time course of positive mood.

The overarching goal of the current study was to address these important gaps in the literature by probing the acute and sustained affective and neural responses to naturalistic positive mood induction, as well as their spontaneous fluctuations during subsequent resting states. To achieve this goal, we developed a novel task involving humour processing and delivery of self-referential positive feedback (Fig. 1). During an acute phase, mood was induced naturalistically and implicitly (that is, with participants unaware of the purpose of the manipulation) by providing participants positive social feedback related to their task performance, which is a potent elicitor of positive affective state^7^. The sustained effect of the manipulation was assessed half an hour later, while participants completed a similar task devoid of any feedback. In addition, spontaneous affective and neural fluctuations in the absence of external stimuli were investigated by means of three resting-state scans that were acquired before the acute phase (rest 1), following the acute phase (rest 2) and following the sustained phase (rest 3; Supplementary Fig. 1). These successive rest and activation runs were interspersed with six mood ratings (Supplementary Methods). Finally, causal interactions between acute neural response to positive mood induction and DMN resting-state connectivity patterns were investigated using spectral dynamic causal modelling spectral-DCM method for resting state functional magnetic resonance imaging (fMRI) data^8^,^9^. Given the potency of social feedback and the established role of VS in mediating the acute responses to pleasurable stimuli, we hypothesized that the delivery of positive social feedback would yield substantial increase in positive mood, which in turn would be associated with VS activation. Furthermore, we expected that neural interactions between VS and DMN during subsequent rest would be linked to sustained positive mood following the offset of the positive stimuli.

To further test our hypotheses, in addition to healthy controls, we included euthymic individuals with a history of recurrent major depressive disorder (MDD)—a prevalent mood disorder that affects millions of individuals worldwide. Owing to the fact that positive mood disturbances are core characteristics of MDD that often persist following remission, the inclusion of a clinical sample allowed us to test fundamental questions about how mood disorders might modulate the neural substrates of mood regulation. Notably, both currently depressed and remitted individuals with a history of depression exhibit attenuated acute response to positive information^10^,^11^, as well as reduced preservation of positive affect in the absence of external stimuli^12^,^13^. Similarly, neuroimaging studies in depression have highlighted blunted VS responsivity to positive stimuli in both currently^14^,^15^,^16^ and remitted MDD^17^, but also altered DMN rest connectivity in currently^18^,^19^,^20^ and remitted MDD^21^,^22^. It is thus unclear whether persistent positive mood disturbances in depression are due to failure in the acute response to positive stimuli, or in the ability to sustain positivity following stimulus offset. Accordingly, the second aim of the current study was to compare the acute response and spontaneous fluctuations in positive mood, as well as the neural interactions that contribute to such patterns, between healthy controls and remitted individuals with a history of recurrent MDD (rMDD). Given previous evidence that depression is associated with reduced positive affect and blunted VS responsivity, we hypothesized that rMDD individuals would exhibit reduced ability to experience and sustain positive mood, alongside reduced VS activation. Finally, we expected that mood dysregulation in rMDD would be linked to abnormal neural interactions between the VS and DMN during rest.

We find that positive social feedback induces acute elevation in positive mood and VS activation in both healthy controls and rMDD individuals, yet only controls show sustained positive mood and VS activation. Among controls, sustained positive mood is associated with a shift towards reciprocal corticostriatal effective connectivity at ensuing rest. Corticostriatal pathways, therefore, contribute to the natural time course of positive mood, while disturbances in these neural interactions limit the ability of individuals with a history of depression to sustain positive mood.

## Results

### Affective responses to the positive mood induction

**Figure 1:**
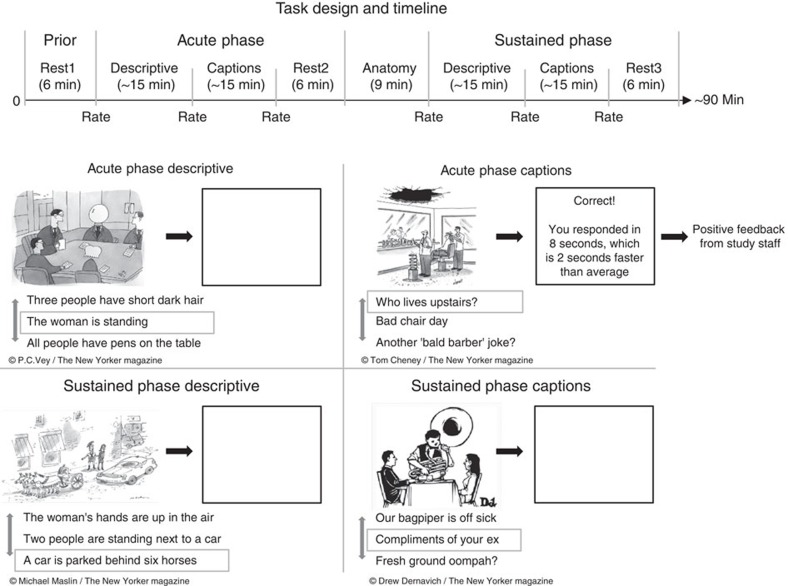
Task design and timeline. The task was composed of four phases: The Acute Descriptive phase, in which participants chose which of three suggested sentences best described a cartoon, without receiving any feedback. The Acute Captions Phase, in which participants chose, for each cartoon, which of three suggested humoristic captions won the New Yorker Caption Contest. In this phase, after 14 of their 18 selections, participants saw a screen indicating that they were correct and that they responded faster than average. Following completion of this phase, participants saw a short pre-recorded video in which an actor that presents himself as the director of research is attributing participants' excellent performance in the caption task to their evidently high levels of emotional intelligence and sense of humour (Supplementary Movie 1). Next, the Sustained Descriptive Phase and Sustained Captions Phase were presented, which are identical to the Acute Descriptive and Captions phases, respectively, with the exception that no feedback was given to participants at any point. In addition, three resting state scans were acquired (before the acute phase (rest 1); following the acute phase (rest 2); and following the sustained phase (rest 3)) to investigate the neural basis of spontaneous affect fluctuations in the absence of external stimuli. Finally, participants were asked to rate their current mood state at six time points throughout the task via a visual analogue mood scale (VAMS).

**Figure 2:**
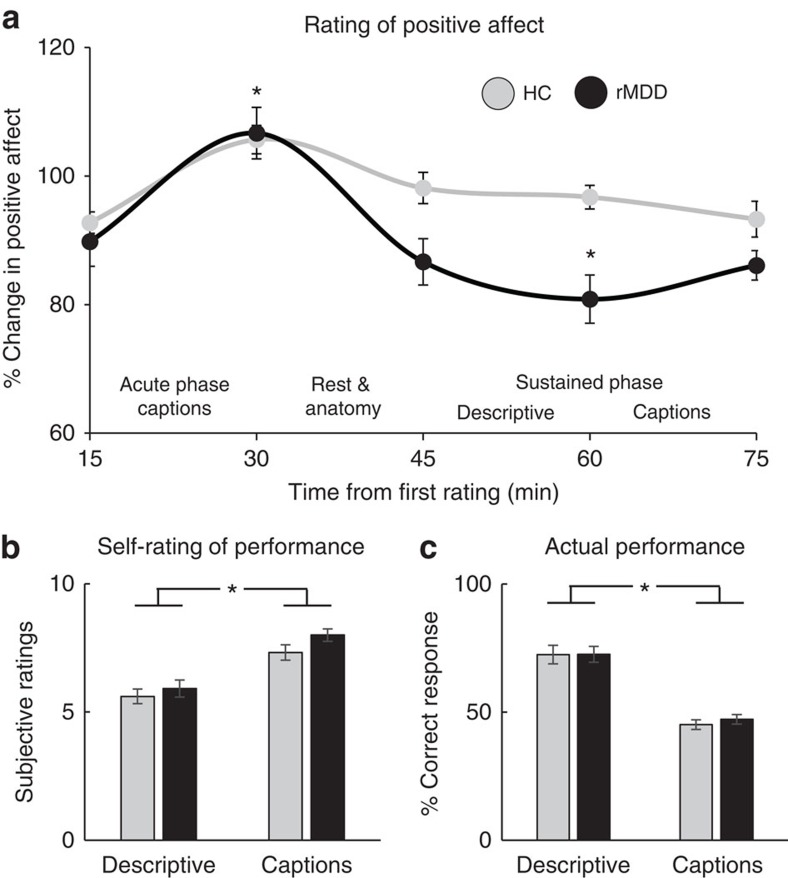
Affective responses to the positive mood induction. (**a**) Mixed ANOVA revealed that both groups exhibited a significant increase in positive mood immediately following the mood induction manipulation (that is, following Acute Phase Captions), yet only controls had a sustained elevation in positive affect over time. Specifically, for controls, but not rMDD, positive mood did not decline for at least 30 min following the mood induction. Mixed ANOVA for self-rating of performance in the task (**b**) and actual performance (**c**) revealed significant differences between the descriptive and captions tasks, although in opposite directions. Participants, across groups, rated their performance to be better in the captions compared with descriptive task, yet their accuracy was actually better in the descriptive compared with the caption task. Taken together, affective responses suggest that participants were engaged in the task and perceived the positive feedback during the captions task as reliably reflecting their performance, which in turn led to a successful induction of positive mood across groups in an ecologically valid and naturalistic way. Bars ±1 s.e.m. *P<0.05. HC, healthy controls (*n*=25); rMDD, remitted individuals with a history of recurrent MDD (*n*=25).

Visual analog mood scale (VAMS) ratings were used to assess participants' mood fluctuations at six time points throughout the study (Fig. 1). Changes in mood throughout the study were calculated separately for each participant as the percentage of change from the first VAMS for each of the five subsequent VAMS ratings thus controlling for in-scanner baseline rating. Those values were entered into a mixed analysis of variance (ANOVA) with Group (Controls versus rMDD) as a between-subject factor, and the five subsequent sampling Time points as a repeated measure. The ANOVA revealed a main effect of Time (*F*_(4,216)_=18.57, *P*<0.001), due to a significant increase in positive mood immediately following the mood induction manipulation (all *P* values <0.001 following the Acute Phase Captions task), as well as a main effect of Group (*F*_(1,54)_=5.30, *P*=0.025), owing to an overall more positive mood in the control compared with rMDD group. Critically, a significant Group by Time interaction also emerged (*F*_(4,216)_=4.08, *P*=0.003), due to more positive mood in controls compared with rMDD 30 min following the offset of the mood induction manipulation (*P*=0.015 following the Sustained Phase Descriptive task). Moreover, as shown in Fig 2a, for controls significant *post hoc* effects revealed higher mood ratings immediately following the mood induction manipulation (following the Acute Phase Captions task) compared with the very first time point (following the Acute Phase Descriptive task; *P*=0.002), and the very last time point (following the Sustained Phase Captions; *P*=0.005). Importantly, however, no differences were found for the control group from immediately following the mood induction manipulation to the subsequent two time points (before and following the Sustained Phase Descriptive task; both *P* values >0.20). Thus, for controls, positive mood did not decline in a significant manner for at least 30 min following the mood induction, highlighting a sustained mood response. In contrast, for the rMDD group, *post hoc* effects included reduced positive affect across all four time points compared with immediately following the mood induction manipulation (all *P* values <0.001 following the Acute Phase Captions task). These results indicate that (1) the novel manipulation successfully induced acute elevation in positive mood across groups, in an ecologically valid and naturalistic way, and (2) positive affect was sustained over time in control but not rMDD individuals (Fig. 2a).

Each trial of the task included a forced choice decision among three options (Supplementary Methods). In light of the fact that there was one correct response for each trial in each phase of the task, participants' actual performance in the task (% of correct response) could be computed. These values were investigated using a mixed ANOVA with Group (Controls versus rMDD) as a between-subject factor, and Condition (Descriptive versus Captions) and Phase (Acute versus Sustained) as repeated measures. Participants' self-ratings of task performance post scan were assessed using a mixed ANOVA with the Group (Controls versus rMDD) as a between-subject factor, and Condition (Descriptive versus Captions) as a repeated measure. Both the ANOVA on actual task performance and self-rating of performance revealed a main effect of Condition (*F*_(1,49)_=75.94, *P*<0.001; and *F*_(1,49)_=104.52, *P*<0.001, respectively), with no Group by Condition interaction (*P*=0.72; and *P*=0.32, respectively). These main effects of Condition were, however, in opposite directions, such that participants in both groups rated their performance as better in the captions compared with the descriptive task, yet they were actually more accurate in the descriptive compared with the caption task (Fig. 2b,c). Notably, across groups, the mean accuracy was 70% and 46% for the descriptive and caption tasks, respectively, that is, significantly above chance level (33%; both *P* values <0.001). Taken together, these findings suggest that participants were engaged in the task and perceived the positive feedback during the captions task as reliably reflecting their performance. Importantly, across the four task types (Acute Phase Descriptive; Acute Phase Captions; Sustained Phase Descriptive; and Sustained Phase Captions) groups did not differ with regard to the time it took participants to make a selection in each trial (reaction time), the number of trials without a selection and the total number of cursor movement (Supplementary Table 1). Similarly, post-scan performance on Penn's Humor Appreciation Test, a computerized test comprised of captionless cartoons and headlines to assess level of humour appreciation^23^, revealed no effect of Group (*F*_(1,49)_=1.61, *P*=0.21). Thus, our findings were not affected by group differences in task difficulty and/or humour comprehension skills.

### Acute neural responses to the positive feedback

**Figure 3:**
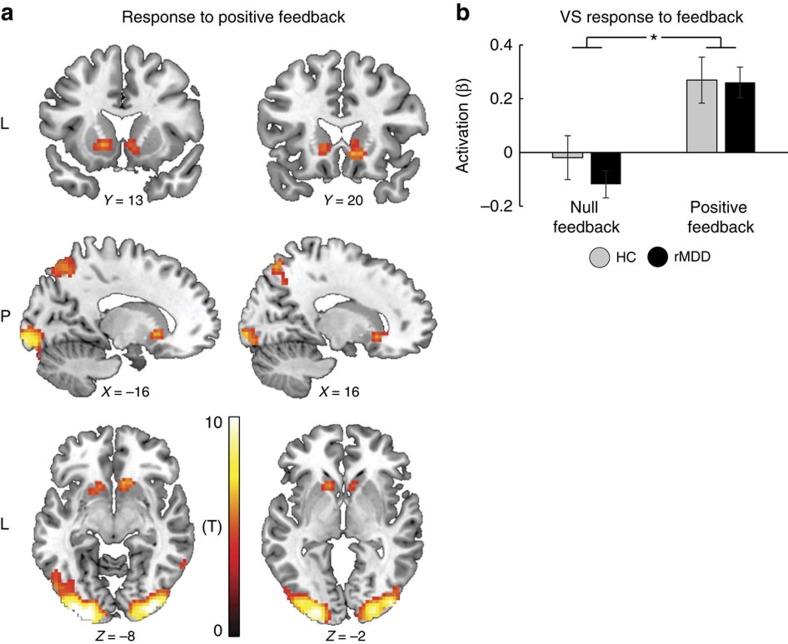
Acute neural responses to the positive feedback. (**a**) A whole-brain analysis across groups revealed increased activation in bilateral VS as well as in visual occipital areas in response to the positive feedback compared with null feedback at a significance level adjusted such that Type I error was controlled for all voxels in the brain using the false discovery rate (FDR), with *q*=0.05 in more than 10 contiguous voxels. (**b**) Mixed ANOVA revealed that both groups responded to the positive feedback with increased VS activation. Bars ±1 s.e.m. *P<0.05. HC, healthy controls (*n*=25); rMDD, remitted individuals with a history of recurrent MDD (*n*=25); VS, ventral striatum.

Whole-brain analysis across groups revealed increased activation in bilateral ventral striatum (VS) as well as in visual occipital areas in response to the positive feedback compared with null feedback (Fig. 3a and Table 1). *Post hoc* analyses of bilateral VS activation in response to positive feedback were investigated using a mixed ANOVA with Group (Controls versus rMDD) as a between-subject factor, and Condition (Positive feedback versus Null feedback) and Side (Left versus Right) as repeated measures. The ANOVA revealed the expected main effect of Condition (*F*_(1,48)_=91.04, *P*<0.001), stemming from an overall increased VS activation to positive compared with null feedback, with no main effects of Group (*P*=0.57), Side (*P*=0.52) or Group by Condition interaction (*P*=0.26). Thus, both groups responded to the positive feedback with acute increase in bilateral VS activation (Fig. 3b). Supportive evidence for those findings also emerged when we subtracted VS activation in response to the positive feedback from its activation in response to null feedback. Specifically, a one-way ANOVA on bilateral VS activation in response to positive minus null feedback yielded no main effect of Group (*P*=0.21).

### Neural responses to the captions task

**Figure 4 f4:**
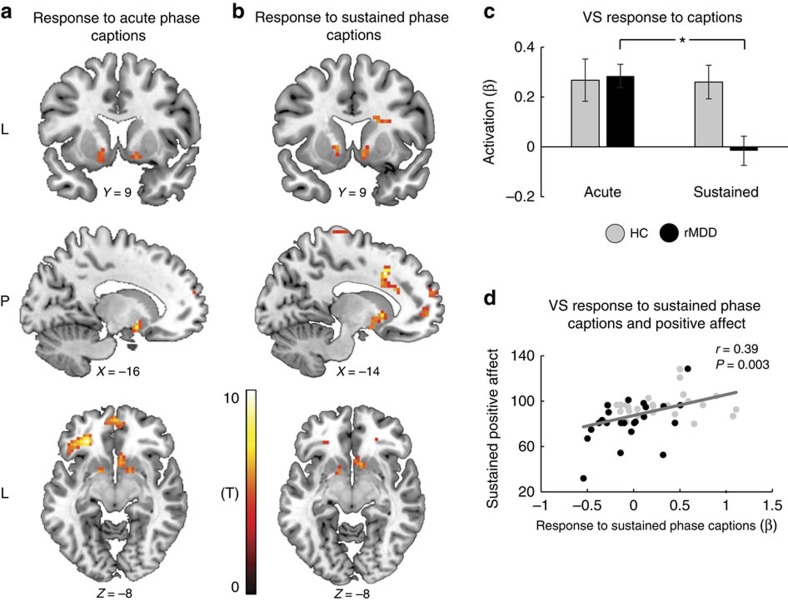
Neural responses to the captions task. Whole-brain analyses across groups revealed bilateral VS activation in response to the captions task compared with the descriptive task, at both the acute (**a**) and sustained (**b**) phases, at a significance level adjusted such that Type I error was controlled for all voxels in the brain using the false discovery rate (FDR), with *q*=0.05 in more than 10 contiguous voxels. Additional activations were found in language and semantic brain regions such as inferior frontal gyrus (IFG), superior temporal gyrus (STG), superior frontal gyrus (SFG) and temporal pole. (**c**) Mixed ANOVA revealed that in the control, but not rMDD group, VS activation in response to the captions task persisted during the sustained phase. For the sake of simplicity, only activations in response to the caption task are presented. (**d**) Across participants, the level of VS activation in response to the Sustained Phase Captions task was positively correlated with mood rating during the sustained phase. Bars ±1 s.e.m. *P<0.05. HC, healthy controls (*n*=25); rMDD, remitted individuals with a history of recurrent MDD (*n*=25); VS, ventral striatum.

Whole-brain analyses separately conducted in the acute and sustained phases for the response to the caption task compared with the descriptive task revealed bilateral VS activation at both phases, alongside activations in language and semantic regions, such as the inferior frontal gyrus, superior temporal gyrus, superior frontal gyrus and temporal pole (Fig. 4a,b and Table 1). *Post hoc* analyses of bilateral VS activation in response to the caption task were investigated using a mixed ANOVA with Group (Controls versus rMDD) as a between-subject factor, and Condition (Caption versus Descriptive), Phase (Acute versus Sustained) and Side (Left versus Right) as repeated measures. The ANOVA revealed a significant Condition by Phase interaction (*F*_(1,48)_=7.14, *P*=0.011), due to overall higher VS activation in the caption relative to the descriptive task during the acute phase. Relevant to the study hypotheses, a significant three-way Group by Condition by Phase interaction also emerged (*F*_(1,48)_=4.32, *P*=0.047), which was attributed to reduced VS activation to captions in the sustained relative to the acute phase only in the rMDD group (*P*=0.013). Thus, for rMDD individuals, but not controls, VS activation in response to the caption task did not sustain (Fig. 4c; for the sake of simplicity, only activations in response to the caption task are presented). Supportive evidence for those findings also emerged when we subtracted VS activation in response to the descriptive task from its activation in response to captions task. Specifically, bilateral VS activation in response to the captions minus descriptive task was investigated using a mixed ANOVA with Group (Controls versus rMDD) as a between-subject factor, and Phase (Acute versus Sustained) as repeated measures. The ANOVA yielded a main effect of Phase (*F*_(1,54)_=10.56, *P*=0.002), due to overall higher VS activation in the acute relative to the sustained phase. Critically, the Group by Phase interaction was also significant (*F*_(1,54)_=4.91, *P*=0.031), due to reduced VS activation to captions minus descriptive task in the sustained phase relative to the acute phase only in the rMDD group (*P*=0.003). Finally, regression analyses across all participants highlighted a significant positive correlation between VS activation in response to captions in the sustained phase and positive mood during the sustained phase (*r*=0.39, *P*=0.003; Fig. 4d). Thus, higher VS activation in the sustained phase was associated with more positive mood during that same time period.

### Corticostriatal effective connectivity at rest

**Figure 5:**
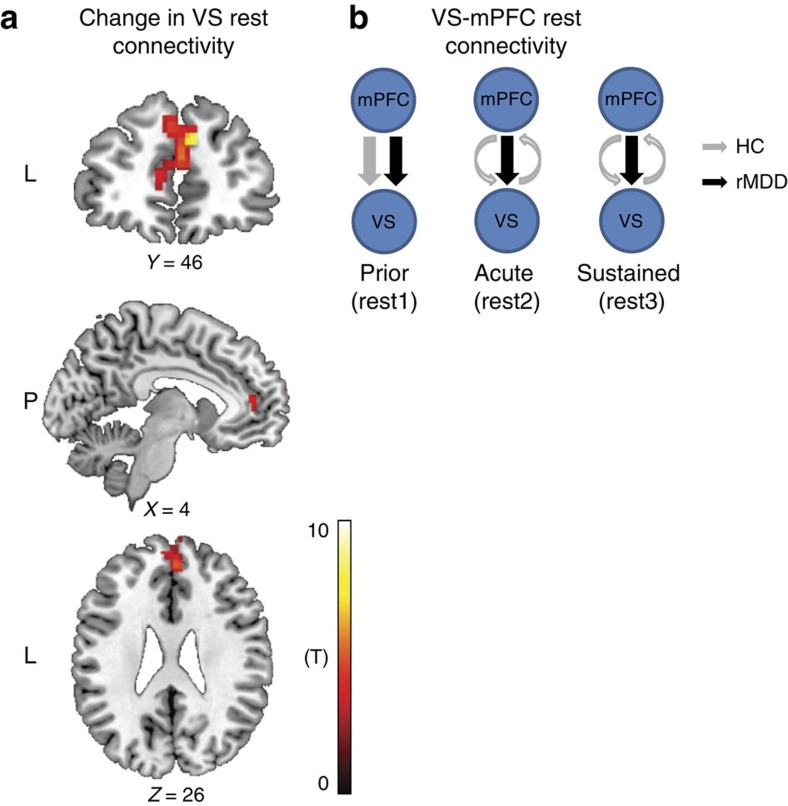
Corticostriatal effective connectivity at rest. (**a**) A single cluster within the DMN, located in the mPFC (226 voxels; MNI peak coordinates: *X*=10, *Y*=50, *Z*=32; *Z*_score_=4.34; *P*_corrected_=0.045) exhibited a significant Group by Time (prior, following acute and following sustained) interaction with the VS time course at a significance level adjusted such that Type I error was controlled for all voxels in the DMN using the false discovery rate (FDR), with *q*=0.05 in more than 10 contiguous voxels. (**b**) Spectral-DCM analyses revealed that, before the mood induction (rest 1), the best fitting model for both groups was the one describing connectivity from the mPFC to the VS. Following the mood induction (rest 2 and rest 3), controls exhibited a change in their mPFC–VS effective connectivity pattern towards a more reciprocal relation, whereas rMDD maintained their original connectivity pattern throughout. HC, healthy controls (*n*=25); mPFC, medial prefrontal cortex; rMDD, remitted individuals with a history of recurrent MDD (*n*=25); VS, ventral striatum.

Resting-state analyses were focused on changes over time in DMN connectivity with regions that acutely responded to the positive mood induction. First, functional connectivity analyses revealed a single cluster within the DMN, located in the medial prefrontal cortex (mPFC), which exhibited a significant Group by Time interaction with the VS time course (Supplementary Methods; Fig. 5a). Next, spectral-DCM was implemented to assess the causal relationship within VS–mPFC connectivity across groups in each of the three resting-state scans. Specifically, three potential causal models were compared using Bayesian model selection: (1) connectivity from the mPFC to the VS (mPFC→VS); (2) connectivity from the VS to the mPFC (mPFC←VS); and (3) reciprocal mPFC–VS connectivity (mPFC↔VS). Bayesian model selection yields a measure of model posterior, ranging from 0 to 1, to indicate the probability of a particular model being the best compared with any other model given the group data^24^. Effect sizes for the best fitting model per group per time point were calculated using Bayesian parametric averaging, thus representing average parameters across the models without any regard for the probability of the models themselves. For further details see Supplementary Methods, as well as refs 8, 9. These analyses revealed that, for both groups, before the mood induction (rest 1), the best fitting model was the one in which the direction of connectivity was from the mPFC to the VS (mPFC→VS), with a posterior probability of 0.968 and effect size of 0.773 for the control group and a posterior probability of 1 and effect size of 0.986 for the rMDD group. Posterior probability of the other two models (mPFC←VS; mPFC↔VS) was close to zero in both groups at rest 1 (Fig. 5b and Table 2). Crucially, in the resting-state scan that immediately followed the acute mood induction (rest 2), controls exhibited a change in their corticostriatal effective connectivity pattern such that the best fitting model was the one with a reciprocal connection (mPFC↔VS), with a posterior probability of 0.957 and effect size of 0.493. The same reciprocal model was also the best fitting model for controls in the third resting-state scan following the sustained phase (rest 3), with a posterior probability of 0.954 and effect size of 0.629. In rMDD, despite an acute increase in positive mood, corticostriatal effective connectivity pattern did not change, maintaining a fixed model of connectivity from the mPFC to the VS (mPFC→VS) following both the acute and sustained phases (posterior probabilities of 1 and effect sizes of 0.781 and 0.779, respectively; Fig. 5b and Table 2).

Notably, differences in Bayesian model comparison do not constitute a formal test of a Group by Time interaction in terms of effective connectivity. Therefore, to confirm the results of the Bayesian model comparison, we analysed the Bayesian model average strengths of the two connections that emerged ((mPFC→VS); (mPFC←VS)) in a mixed ANOVA with Group (Controls versus rMDD) as a between-subject factor, and Direction ((mPFC→VS) versus (mPFC←VS)), and Time (three resting state) as repeated measures. As expected, the ANOVA revealed a significant Group by Time interaction (*F*_(2,88)_=10.29, *P*<0.001), due to a group difference in connectivity magnitude at the second (*P*=0.012) and third (*P*<0.001), but not first, resting-state scan (*P*=1). In addition, a significant three-way Group by Direction by Time interaction also emerged (*F*_(2,88)_=5.66, *P*=0.005), which was attributed to reduced (mPFC→VS) connectivity strength in the control relative to rMDD group at the second (*P*=0.012) and third (*P*<0.001), but not first, resting-state scan (*P*=0.92; Supplementary Fig. 2a). Moreover, controls exhibited increased (mPFC←VS) connectivity relative to rMDD at the second (*P*=0.017) and third (*P*=0.009), but not first resting-state scan (*P*=0.97; Supplementary Fig. 2b).

## Discussion

The goal of the current study was twofold. First, we aimed to investigate whether acute neural responses to positive stimuli interact with the DMN at subsequent rest, and evaluate the potential contribution of such neural interactions to the natural time course of positive mood fluctuations. Second, we compared the acute response and spontaneous fluctuations in positive mood and their underlying neural substrates between healthy controls and remitted individuals with a history of MDD. Findings on the affective ratings confirmed that our novel task successfully induced positive affect across groups in an ecologically valid and naturalistic way, and furthermore, that such positive mood sustained over time in controls, but not rMDD individuals. fMRI data revealed that VS activation patterns mirrored positive mood fluctuations, as it acutely increased in both groups, showed sustained activation in the absence of positive feedback among controls, but dissipated quickly in the rMDD group. Finally, spectral-DCM analyses indicated that, among controls, sustained positive mood was associated with a shift in resting state–directed effective connectivity towards a more reciprocal relationship between the VS and mPFC, a shift that was absent in the rMDD group. Collectively, these findings suggest that corticostriatal pathways contribute to the natural time course of positive mood fluctuations and that disturbances of those neural interactions may characterize individuals with a past history of mood disorders.

Previous attempts to experimentally induce positive mood achieved only partial success^1^. Here, in order to maximize the effectiveness of the mood manipulation, we choose to implement positive social feedback oriented towards the self, which is a potent elicitor of positive affect^7^. The delivery of positive social feedback without participants' explicit awareness of the purpose of the manipulation was designed to allow a naturalistic induction of positive mood. Indeed, across both groups, participants' post-scan subjective assessments and VAMS ratings indicated that the positive feedback was perceived as reliable and yielded a significant elevation in positive mood (Supplementary Table 2). Importantly, the acute effect of the positive mood induction was assessed only once via VAMS rating, following both the task-related positive feedback and the positive social feedback that was delivered during the subsequent Skype call. Therefore, we cannot determine whether acute elevation in positive mood was the result of one of these feedback types, or the combination of the two. Regardless, to the best of our knowledge, this is one of the first demonstrations of a successful naturalistic induction of positive mood within the constraints of the neuroimaging environment. By doing so, we were able to associate the response to the positive feedback with robust VS activation. Similar increases in VS activation were previously demonstrated in response to various positive stimuli including pleasurable food^25^, music^26^ and sights^27^, as well as monetary gain^28^. Therefore, and in accordance with the well-established role of the VS in mediating the response to positive stimuli^2^,^3^, the current increase in VS activation in response to the positive feedback may relate to the rewarding properties of such feedback.

Increased VS activation was also found in response to the caption task itself, alongside activations in semantic frontal and temporal regions. These activations are consistent with the findings of previous studies examining the neural substrates of humour comprehension, and are thought to sub-serve two fundamental processes of humour comprehension^29^. Specifically, semantic regions are considered particularly relevant for the detection and resolution of incongruity, while VS function is tied with the consequent elicitation of positive feeling^29^. Notably, VS activation in response to the caption task was found at both the acute and sustained phases, yet mood elevation was only evident at the acute phase. VS activation in response to the caption task may, therefore, reflect some other components besides positive mood processing. For example, striatal activation was recently linked to cognitive control of memory retrieval^30^, particularly the recall of positive memory^31^, as well as gating during selection from working memory^32^, all of which are relevant processes for the completion of the captions task.

While VS involvement in the acute response to positive stimuli is supported by previous work, the current novel design enabled us to explore the potential effect of such acute response on neural fluctuations following stimulus offset. To this end, effective connectivity analyses revealed that sustained positive mood in the absence of external stimuli in healthy controls involved a shift towards a more reciprocal relationship between the VS and a single cluster within the DMN located in the mPFC. In other words, whereas resting VS–mPFC connectivity before the mood induction was solely driven by the mPFC, a reciprocal (bidirectional) VS–mPFC functional connection emerged following the mood induction and persisted throughout the study. Crucially, since our regions of interest included only the VS and mPFC, we cannot rule out the potential contribution of additional brain regions in sustained positive mood. Nevertheless, these novel findings concur with extensive human and nonhuman work highlighting the pivotal role of functional interactions between the VS and mPFC in reward processing^33^,^34^,^35^. Directly relevant to the current findings, VS and mPFC activations were recently associated with processing the self-relevance of positive social feedback and the integration of such feedback into the self-concept^36^. Moreover, during rest, DMN function, and particularly within the mPFC, is believed to support self-referential affective processes^4^,^5^,^6^. On the basis of this prior literature, we suggest that neural interactions within corticostriatal pathways may contribute to the integration of acute positive information into self-concept, thus increasing its significance and prolonging its affective impact. In future studies, it would be interesting to investigate whether other sustained mood states (for example, sadness) rely on a shift in DMN resting-state connectivity with different brain regions, particularly regions that acutely respond to the sadness-inducing stimuli.

Sustained sad mood and disturbances in positive mood are core characteristics of MDD that tend to persist following remission. Interestingly, we found that the acute response to the mood manipulation of rMDD individuals was comparable to the response of healthy controls, manifested as elevation in positive mood and VS activation in response to positive feedback. At first, normative acute response to positive stimuli in rMDD may appear surprising given ample evidence for blunted behavioural^10^,^11^ and neural^14^,^15^,^17^ reactivity to positive stimuli in depression. However, previous work has shown that currently depressed individuals cannot achieve or sustain equivalent levels of positive affect^12^, while studies assessing individuals with subsyndromal depression^13^,^37^ found behavioural deficits that specifically emerged as reduced preservation of positive affect over time. Here as well, despite comparable acute elevation in positive affect, individuals with a history of depression were unable to sustain positive mood over time, even years after the last major depressive episode, and in the absence of current depressive symptoms (the current rMDD sample was in remission, on average, since 2.8 years and groups did not differ in their depressive symptoms; Table 3). Collectively, these findings suggest that individuals with increased risk for future depression (owing to current sub-clinical depressive symptom^38^ or a history of depression^39^) are characterized by an inability to sustain positive mood following stimuli offset, rather than a failure in acute responses to positive stimuli. Such insights may have clinical implications by suggesting that treatments, many of which primarily focus on negative thinking, should also target strategies for enhancing sustained positive affect^40^. Indeed, depressed patients often link their remission to reinstatement of positive feelings such as optimism and self-confidence^41^.

Inability to sustain positive mood in rMDD was associated with reduced VS activation over time and a lack of mood-induced shift in VS–mPFC resting-state connectivity. Both reduced VS activation in response to reward^17^, and abnormal DMN resting-state connectivity^21^,^22^, have been reported before in remitted individuals, even in the absence of current depressive symptoms, and are thought to represent markers for increased vulnerability to experience future depressive episodes. In currently depressed individuals, reduced VS activation in response to reward^16^, and inability to sustain VS activation while explicitly attempting to upregulate positive affect^42^, have been associated with reduced intensity of positive affect. In addition, increased ability to sustain VS activation following 2 months of antidepressant treatment predicted increased positive affect^43^. Thus, reduced ability to sustain VS activation over time in rMDD may relate to their inability to sustain positive mood. Indeed, regression analyses highlighted a significant positive correlation between VS activation in response to captions in the sustained phase and positive mood during the sustained phase. Nevertheless, as indicated above, it is important to note that VS activation in response to the caption task may not relate exclusively to mood.

In resting-state studies, depression has been consistently associated with enhanced DMN interconnectivity and dominance of DMN over task-positive function, which in turn were interpreted to reflect biases towards internal ruminative thoughts at the cost of reduced engagement with external stimuli^18^,^19^. Such pattern of DMN connectivity in depression may also account for our findings in rMDD of diminished capacity to establish reciprocal connectivity between the DMN and signals from non-DMN regions as the VS during rest. Importantly, exploratory analyses examining patterns of connectivity between the VS and the entire DMN for every participant for each of the seven phases of the study (three resting-state and four task phases) revelled a main effect of Phase (*F*_(6,264)_=10.16, *P*<0.001), due to stronger VS–DMN functional connectivity during the four tasks compared with during the three resting-state scans across groups, but no effect of Group (*F*_(1,44)_=0.41, *P*=0.84) or a Group by Phase interaction (*F*_(6,264)_=0.15, *P*=0.99; data not shown). Taken together, VS connectivity with the entire DMN did not differ across groups nor was it impacted by the mood manipulation. Disturbances specifically within corticostriatal pathways may, therefore, disrupt the natural time course of positive mood fluctuations in depression, potentially by limiting individuals' ability to reflect on the self-relevance of a preceding positive feedback when left with their thoughts. While we can only speculate about mentation during the resting-state scan, these conjectures are in line with cognitive theories of depression that attribute blunting of positive affect to spontaneous thought processes such as rumination that occur in the absence of significant external emotional stimuli^44^. More research is warranted to investigate whether such deficits represent a trait-like vulnerability factor or a ‘scar' following previous depressive episodes.

In summary, using a novel paradigm involving humour processing and positive social feedback, we were able to naturalistically induce positive mood, which was associated with acute elevation in VS activation in both healthy controls and remitted individuals with a history of depression. Only in controls, however, such positive mood and VS activation were sustained over time. Moreover, controls, but not rMDD individuals, exhibited a shift towards reciprocal corticostriatal effective connectivity during ensuing resting states. Corticostriatal pathways, therefore, play an important role in the natural time course of positive mood fluctuations, while disturbances in these neural interactions may limit the ability of individuals with a history of depression to sustain positive mood, which might increase risk for relapse.

## Methods

### Participants

Thirty never depressed healthy controls and 30 remitted individuals with a history of rMDD were recruited from the community. Groups were matched for age, sex, ethnicity, handedness, education and annual income (Table 3). Exclusion criteria for controls included: any Diagnostic and Statistical Manual (DMS-IV) psychiatric diagnosis based on a structured clinical interview^45^, use of psychotropic medication and the presence of first degree relatives with psychiatric illnesses. Controls were also excluded if they had dementia, a history of seizures, hypothyroidism, severe concussion, loss of consciousness longer than 2 min or if they tested positive for drugs or pregnancy in urine screening. Similar criteria were applied to rMDD participants, except that they (1) met criteria for at least one major depressive episode in the past 5 years and had been in remission for at least 8 weeks before testing, (2) were permitted to have used psychotropic medication in the past if the appropriate washout period had passed and (3) were permitted to have past diagnoses of anxiety disorders if secondary to depression. In addition, on the morning of the study visit, all participants completed the 21-item Beck Depression Inventory (BDI-II) to assess current depression severity^46^, and the Snaith-Hamilton Pleasure Scale (SHAPS) to assess anhedonia levels^47^. Mean BDI scores for the control and rMDD groups were 1.23±2.08 and 2.17±2.46, respectively (*F*_(1,48)_=2.12, *P*=0.15). BDI Scores ranged from 0 (reported by *n*=16 or 61.5%) to 8 (reported by *n*=1 or 3.8%) in the control group, and from 0 (reported by *n*=10 or 38.4%) to 9 (reported by *n*=1 or 3.8%) in the rMDD group, confirming full remission (Table 3). All participants received payment for their time and provided written informed consent to a protocol approved by the Committee on the Use of Human Subjects in Research at McLean Hospital.

### Procedure and task

Participants were brought into the scanner after signing consent and MRI screening forms, as well as completing the BDI^46^ and SHAPS^47^ questionnaires. The functional scan started with a 6-min long resting-state scan during which participants saw a black fixation cross on a white screen and were asked to lay still with their eyes open (rest 1). Next, in-scanner baseline mood ratings were assessed via a VAMS asking participants to rate how they felt in the moment, ranging from very negative to very positive. Participants were then told that they were about to be presented with several tasks. In the first task, a cartoon was presented on the screen alongside three short descriptive sentences (Acute Phase Descriptive; Fig. 1). One of the three sentences (randomly selected) was marked with a cursor. Participants were instructed to move the cursor using a response box and to select which of the three sentences best described the cartoon, as quickly and accurately as possible. They were further informed that they would not receive any feedback on their performance. Each cartoon and three sentences were presented until the participant made a selection or up to 18 s in case no selection was made, and were followed by a 6-s long empty screen (null feedback). The task included a total of 18 such trials, which were separated into two sections of 9 trials with a short break in-between, for a total length of ∼15 min. After task completion, a second VAMS rating was administrated.

Next, participants saw the instructions for the second task, again featuring cartoons, only this time the three descriptive sentences were replaced with three humorous captions (Acute Phase Captions; Fig. 1). All cartoons and captions were taken from the New Yorker magazine Cartoon Caption Contest, a contest in which readers are asked to choose which of the three suggested captions is the funniest with respect to a given cartoon. Participants were made aware of this, and their task, accordingly, was to choose for each cartoon which of the three captions won the New Yorker Caption Contest. The overall design of the caption task was identical to the descriptive one, only this time participants were told that they may receive feedback on their performance. Indeed, regardless of their accuracy, participants were presented in 14 of the 18 trials with a screen indicating that they were correct (positive feedback) following their selection of a specific caption. Positivity and believability of the feedback were further enforced by including participants' actual response time in the feedback screen after each trial, and indicating that they were faster than prior participants by a random number of seconds (1–3±1). No feedback was given if no selection was made, and no feedback on response time was given if it was slower than the participant's mean+2 s.d. until that point. Moreover, on task completion, participants were told by study staff that their performance in the caption task was well above average and that this was brought to the attention of the research director who would like to briefly share his impression with them. At this point, while lying in the scanner, participants saw a short pre-recorded video that started with the launch of a Skype call (the pointer of a computer mouse is seen crossing the computer screen to start a Skype call and a Skype ringtone is heard after call initiation), which was answered by an actor presenting himself as the director of research. Within the 90-s long pre-recorded video the actor attributes participants' excellent performance in the caption task to their evidently high levels of emotional intelligence and sense of humour (Supplementary Movie 1).

Next, participants completed a third VAMS rating and underwent a second 6-min long resting-state scan (rest 2), as well as a 9-min long anatomical scan, for the total length of ∼15 min. Following the anatomical scan, a fourth VAMS rating was performed. Participants then completed the sustained descriptive task, fifth VAMS, and the sustained caption task, which were identical to the respective acute tasks with the exception that this time no feedback was provided at any point. Next, following a sixth VAMS rating, the third and final resting-state scan was acquired (rest 3). See Supplementary Fig. 1 for a full graphical description of the order of the interleaved tasks and resting-state scan throughout the study. Post scan, participants rated their performance in the descriptive and caption tasks on a scale of 1 (very poor) to 10 (very good), and completed Penn's Humor Appreciation Test, a computerized test comprised of captionless cartoons and headlines, to assess the level of humour appreciation^23^.

Several criteria were required to reliably and effectively convey positive feedback. First, the task needed to address a skill for which people are uncertain about their level of performance (so that the positive feedback could be plausible), and second, feedback needed to reflect participants' ability to perform the task (that is, success was not just due to luck). Importantly in this regard, the two distractors that were presented alongside the winning caption for every cartoon were taken from the New Yorker magazine pool of alternative captions, thus representing suggestions that were relevant in the context of a given cartoon, yet were ruled less favourably by the New Yorker magazine editors. Therefore, all three captions for a given cartoon presented plausible options, allowing us to deliver reliable positive feedback regardless of the actual choice (similarly, in the descriptive task, the two distractor sentences alluded to information that appeared in their associated cartoon yet contained an error, for example, an incorrect number of items than what was shown in the cartoon). Indeed, when asked after study completion whether they perceived the positive feedback as reliable, and if so how did it make them feel, participants' responses were all positive, and included statements such as: ‘Made me feel like I have a good sense of humour and can read into things pretty well', ‘It felt good', ‘Motivated me to continue performing well', ‘It made me feel pretty confident', ‘It made me feel proud about myself', ‘Made me more positive', ‘It was very uplifting and encouraging', ‘Showed that I was doing the task correctly', ‘I felt good about it and surprised', ‘ It made me smile', ‘Excited and happy'. See Supplementary Table 2 for a full list of participants' responses.

The caption task itself was centred on humour comprehension processes (matching a caption to a humorous cartoon). This was done in order to investigate neural response to positive stimuli^29^, independently of the mood manipulation (the positive feedback), thus enabling the comparison of the acute and sustained phases. Overall, a total of 72 cartoons were presented to participants throughout the scan (18 × 4). Before the current study, an independent sample of healthy adults (*n*=15) rated the valence and arousal of all cartoons (when appearing without the captions) using self-assessment manikin scales. Those ratings were then used to counterbalance cartoons across the four tasks, as well as to exclude the cartoons that were rated in the top 10% for either valence or arousal to avoid humour comprehension process in the descriptive phases. Consistency was also maintained with respect to the length of the descriptive sentences and humoristic captions across the tasks. With regard to task performance in the current sample, no group difference emerged with respect to: the length of time needed to make a selection (controls: 10.48±2.15 versus rMDD: 9.78±2.46 s; *F*_(1,49)_=1.83, *P*=0.183), the number of trials without a selection (out of 18; controls: 1.31±1.70 versus rMDD: 0.97±1.92; *F*_(1,49)_=0.74, *P*=0.395) or the total number of cursor movements needed to make a selection (controls: 1.31±0.45 versus rMDD: 1.46±0.64; *F*_(1,49)_=1.18, *P*=0.283; see Supplementary Table 1 for a full description of group average reaction time, number of trials without a selection and total number of cursor movement in each of the four task types). Finally, after study completion, all participants indicated that they were not familiar with any of the cartoons and/or captions that they saw during the task.

### fMRI data acquisition

MRI scanning was conducted using a Siemens Tim Trio 3T MR scanner with a 32-channel head coil. For each of the four tasks, 360 functional volumes were acquired using a T2-weighted spin echo planar imaging sequence (repetition time=2,500 ms; echo time=29 ms; field of view=212 mm; matrix=64 × 64; resolution=3.3 × 3.3 × 2 mm^3^; 48 contiguous slices aligned to the AC–PC plane). For the three resting-state scans the same parameters were used to acquire 144 functional volumes. High-resolution T1-weighted MPRAGE images were also acquired (repetition time=2,530 ms; echo time=1.64 ms; field of view=256 mm; matrix=256 × 256; resolution=1 × 1 × 1 mm^3^; 176 slices).

### fMRI data analyses

fMRI task and resting-state data were preprocessed using Statistical Parametric Mapping (SPM12; Wellcome Department of Cognitive Neurology, London, UK; http://www.fil.ion.ucl.ac.uk/spm/) and included realignment and geometric unwarping of echo planar images using magnetic fieldmaps, coregistration of functional and anatomical images, segmentation, nonlinear volume-based spatial normalization (Montreal Neurological Institute (MNI) template) and spatial smoothing with a Gaussian filter (6 mm (full-width at half-maximum)). Additional software (Artifact Detection Tools (ART); http://web.mit.edu/swg/software.htm) was used to identify and exclude outliers in the global mean image time series (threshold: 3 s.d. from the mean) and movement (threshold: 1 mm; measured as scan-to-scan movement, separately for translation and rotation) parameters.

For task-related data, hemodynamic responses were modelled using a canonical hemodynamic response function and convolved with onset times of task regressors to form the general linear model (GLM) at the single-subject level. Regressors were separately defined for each of the four task sections (Acute Phase Descriptive; Acute Phase Captions; Sustained Phase Descriptive; and Sustained Phase Captions). Regressors included: (1) response to the task, from the appearance of the cartoon and sentences or captions until participants' first move of the cursor. (2) Movement, from participants' first move of the cursor until a selection was made and (3) Response to the feedback, which can be either null feedback in the Acute Phase Descriptive task and the two sustained phases, or positive feedback in the Acute Phase Captions task. GLMs also included high-pass temporal filtering (0.008 Hz), as well as nuisance regressors accounting for trials in which no selection was made, outlier time points and six rigid-body movement parameters. Analyses were focused on three contrasts: (1) Response to positive feedback in the Acute Phase Captions compared with response to null feedback in the Acute Phase Descriptive, (2) Response to the task in the Acute Phase Captions compared with response to the task in the Acute Phase Descriptive and 3) Response to the task in the Sustained Phase Captions compared with response to the task in the Sustained Phase Descriptive. Single-subject whole-brain activation maps in response to these contrasts were used to separately construct three second-level random effects analyses (see statistical analyses section).

Resting-state data were analysed using the CONN toolbox (http://web.mit.edu/swg/software.htm). Seed-to-voxel connectivity module in CONN toolbox follows the standard procedure of first transforming the correlation coefficient values (*r*) into *z*-scores using the Fisher's *r* to *z* transformation, and then testing whether the difference in *z*-transformed correlation coefficients is different from zero^48^. Using the CONN toolbox, first-level analyses of preprocessed functional and structural images included high-pass (0.008 Hz) and low-pass (0.09 Hz) temporal filtering, as well as accounting for cerebrospinal fluid (CSF) and white matter (WM) mean signals, outlier time points and six rigid-body movement parameters. Second-level analyses were focused on changes over time in DMN resting-state connectivity with regions that acutely responded to the positive mood induction. To this end, using the CONN toolbox Second-Level Results Seed-to-Voxel module, the bilateral VS (as defined from the whole-brain analysis of the response to positive feedback, Fig. 3) was used as a seed in a mixed ANOVA with Group (Controls versus rMDD) as a between-subject factor, and resting-state acquisition Time (prior, following acute and following sustained) as a repeated measure. Regions within the DMN are reported significant if a Group by Time interaction with the VS time course emerged (see statistical analyses section). DMN mask was defined by implementing the same ANOVA but using the precuneus as a seed and while exploring connectivity across Group and Time (Supplementary Fig. 3).

Causal interactions during rest between the VS (defined as before, Fig. 3) and mPFC (as defined from functional connectivity analyses, Fig. 5a) were characterized using spectral DCM approach (spectral-DCM), which is especially suited for group comparisons of resting-state fMRI data. In short, DCM is a framework for fitting differential equation models of neural states to neuroimaging data using Bayesian inference. Spectral-DCM, which was recently implemented in the analyses of resting-state fMRI data^8^,^9^, incorporates spontaneous noisy fluctuations into the state equations, thus allowing to model dynamics without any experimental inputs, such as during resting state (for further details see refs 8, 9). Accordingly, using Spectral-DCM analyses, we investigated the direction of connectivity between VS and mPFC across groups and the three resting state time points. Analyses included the following steps: (1) Construction of a first-level GLM for every participant for every resting state time point, which included high-pass (0.008 Hz) temporal filtering, CSF and WM mean signals, outlier time points, and six rigid-body movement parameters. (2) Extraction of the individual fMRI time course from the VS and mPFC masks that were defined from the group contrasts. (3) Specification and estimation of the three potential models per participant per resting state time point. (4) Implementation of a *post hoc* Bayesian model comparison routine to determine the best fitting model per group per time point^8^,^9^,^24^.

### Statistical analyses

From the original sample of 60 participants, 5 participants were excluded due to difficulty in understanding the instructions or making a selection in time, which did not permit delivery of sufficient positive feedback (2 rMDD, 3 controls). Five additional participants were excluded due to technical problems in the delivery of the video or audio components of the feedback (3 rMDD, 2 controls), leaving a total of 25 participants in each group.

In all four whole-brain second-level random effects analyses that are reported throughout the text (Figs 3a, [Fig f4],4a,b Supplementary Fig. 3), significance level was adjusted such that Type I error was controlled for all voxels in the brain using the false discovery rate, with *q*=0.05 in more than 10 contiguous voxels. Similarly, in Fig. 5a significance level was adjusted such that Type I error was controlled for all voxels in the DMN using the false discovery rate, with *q*=0.05 in more than 10 contiguous voxels. For all ANOVA's reported throughout the text *post hoc* effects were pursued using Bonferroni correction.

## Additional information

**How to cite this article:** Admon, R. & Pizzagalli, D. A. Corticostriatal pathways contribute to the natural time course of positive mood. *Nat. Commun.* 6:10065 doi: 10.1038/ncomms10065 (2015).

## Supplementary Material

Supplementary Information Supplementary Figures 1-3 and Supplementary Tables 1-2

Supplementary Movie 1 In order to naturalistically and effectively induce positive mood, participants were told upon task completion that their performance was well above average and that this was brought to the attention of the research director who would like to briefly share his impression with them. At this point, while lying in the scanner, participants saw a short pre-recorded video that started with the launch of a Skype call (e.g., the pointer of a computer mouse is seen crossing the computer screen to start a Skype call and a Skype ringtone is heard after call initiation), which was answered by an actor presenting himself as the director of research. Within the 90-sec long pre-recorded video the actor attributes participants' excellent performance in the caption task to their evidently high levels of emotional intelligence and sense of humour.

## Figures and Tables

**Table 1:** Clusters showing significant response to the task.

**Contrast**	**Region**	**# of voxels**	***X***	***Y***	***Z***	***Z-*****score**	***T value***
Positive versus null feedback	Right ventral striatum	52	9	20	−7	5.65	6.90
	Left ventral striatum	41	−15	14	−4	5.62	6.84
	Right inferior occipital gyrus	496	36	−88	−7	>10	11.84
	Left inferior occipital gyrus	747	−24	−94	−13	>10	15.08
	Right middle occipital gyrus	664	30	−67	35	6.25	8.00
	Left middle occipital gyrus	338	−12	−73	56	5.52	6.67
Acute phase cartoons versus descriptive	Right ventral striatum	34	10	14	−10	3.94	4.33
	Left ventral striatum	26	−12	14	−4	4.26	4.76
	Left orbitofrontal (BA 11)	111	−27	35	−13	6.29	8.02
	Right cingulum (BA 31)	84	3	−16	47	4.59	5.21
	Right Superior temporal gyrus (STG)	223	63	−43	14	4.39	4.94
	Medial orbitofrontal (BA 11)	135	0	56	−10	4.20	4.68
	Right temporal pole	29	45	17	−31	4.16	4.63
Sustained phase cartoons versus descriptive	Right ventral striatum	36	9	14	−7	3.04	3.42
	Left ventral striatum	24	−12	14	−1	3.38	3.90
	Right inferior frontal gyrus (IFG)	205	18	29	14	4.18	5.18
	Left orbitofrontal (BA 11)	70	−27	35	−10	4.13	5.10
	Right cingulum (BA 31)	75	3	−13	47	3.75	4.47
	Right superior frontal gyrus (SFG)	79	−6	65	11	3.33	3.82
	Right superior temporal gyrus (STG)	28	51	−31	2	3.25	3.71
	Left superior parietal	51	−45	−28	62	2.80	3.09

BA, Brodmann area; Montreal Neurological Institute (MNI).

Clusters showing significant response across groups at a significance level adjusted such that Type I error was controlled for all voxels in the brain using the false discovery rate (FDR), with *q*=0.05 in more than 10 contiguous voxels. Coordinates are presented in MNI space.

**Table 2:** Posterior probability measures per group per resting-state scan.

**Group**	**Model**	**Prior**	**Acute**	**Sustained**
HC	mPFC→VS	**0.968 (0.773)**	0.043	0.046
	mPFC←VS	<0.001	<0.001	<0.001
	mPFC↔VS	0.032	**0.957 (0.493)**	**0.954 (0.629)**
rMDD	mPFC→VS	**1.000 (0.986)**	**1.000 (0.781)**	**1.000 (0.779)**
	mPFC←VS	<0.001	<0.001	<0.001
	mPFC↔VS	<0.001	<0.001	<0.001

HC, healthy controls; mPFC, medial prefrontal cortex; rMDD, remitted individuals with a history of recurrent major depressive disorder; VS, ventral striatum.

Posterior probability values can range from 0 to 1 to represent the probability of a particular model being the best compared with any other model given the group data. Values in bold represent Bayesian model selection (BMS) for the best fitting model per groups per resting state scan. Bayesian parametric averaging (BPA) in parenthesis represent effect sizes for the best fitting model per group per time point. Accordingly, controls exhibit a shift towards a reciprocal pattern of corticostriatal effective connectivity following the mood induction, which was sustained over time. In rMDD individuals, despite experiencing an acute increase in positive mood, the best fitting model to account for corticostriatal connectivity did not change over time.

**Table 3:** Demographic and clinical characteristics of remitted individuals with a history of recurrent major depressive disorder (rMDD) and healthy controls (HC).

	**HC Group** (***N*****=30)**		**rMDD Group (*****N*****=30)**		***P*****value**
	**Mean**	**s.d.**	**Mean**	**s.d.**	
Age (years)	30.2	11.1	31.2	12.5	0.73
Education (years)	15.6	2.4	15.4	2.2	0.78
Beck depression inventory (BDI-II)	1.2	2.1	2.2	2.5	0.15
Snaith-Hamilton Pleasure Scale (SHAPS)	20.8	5.9	21.1	5.1	0.85
Number of prior major depressive episodes	—	—	2.4	1.0	
Duration of remission (years)	—	—	2.8	2.4	
	*N*	%	*N*	%	
Female	22	73.3	22	73.3	1.00
Caucasian	25	83.3	21	70.0	0.23
Handedness (right)	30	100	30	100	1.00
Annual income <$50,000	17	56.7	19	63.3	0.61
