# Supplementary material for: Corticostriatal pathways contribute to the natural time course of positive mood
Source: Nat Commun. 2015 Dec 7;6:10065. doi: 10.1038/ncomms10065 (PMC4686763; doi:10.1038/ncomms10065)
Supplement: Supplementary Information — Supplementary Figures 1-3 and Supplementary Tables 1-2 [file ncomms10065-s1.pdf]

**Supplementary Figure 1**

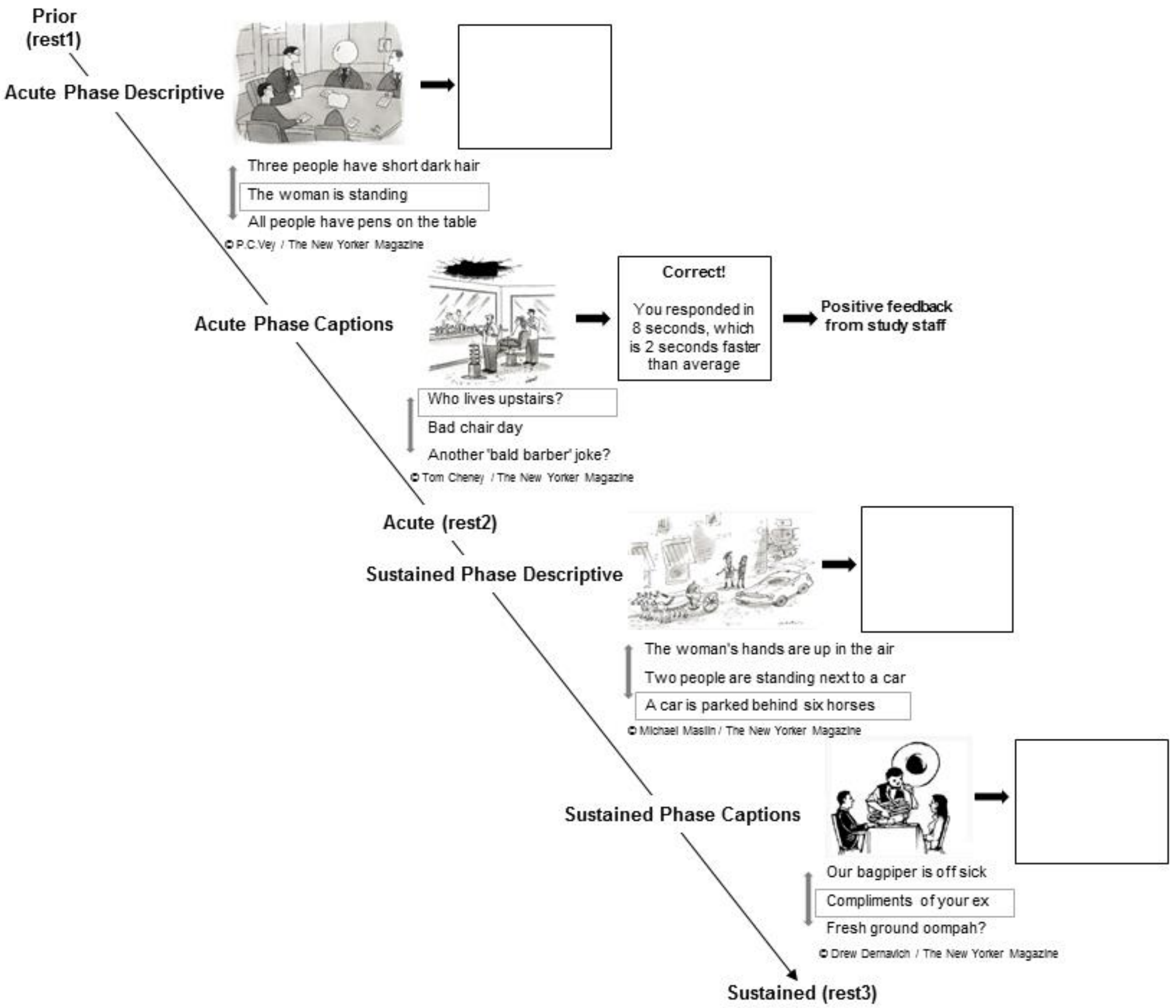


Supplementary Figure 1 - Graphical description of the order of the interleaved tasks and resting state scan throughout the study. In the acute phase, mood was induced naturalistically and implicitly (i.e., with participants unaware of the purpose of the manipulation) by providing participants positive social feedback related to their task performance. The sustained effect of the manipulation was assessed 30 min later while participants completed a similar task devoid of any feedback. In addition, spontaneous affective and neural fluctuations in the absence of external stimuli were investigated by

means of three resting-state scans that were acquired prior to the acute phase (rest1), following the acute phase (rest2), and following the sustained phase (rest3).

**Supplementary Figure 2**

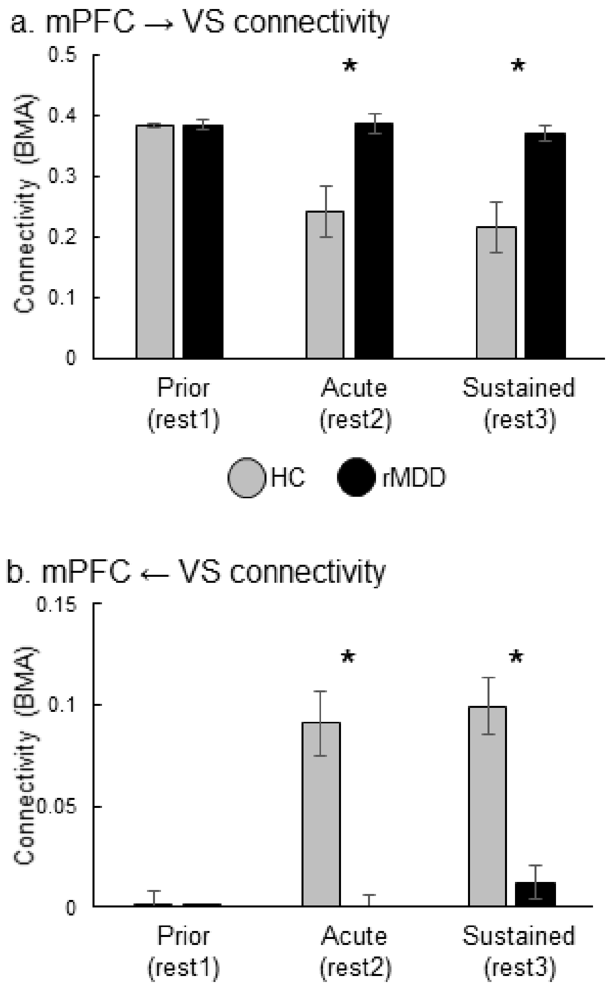


<u>Supplementary Figure </u>2 - Bayesian model averages (BMA) of the (**a**) [mPFC → VS] and (**b**) [mPFC ← VS]) connections per group per resting state time point. Notably, mixed ANOVA revealed that the control group, relative to rMDD group, exhibited reduced [mPFC → VS] connectivity strength but enhanced [mPFC ← VS] connectivity strength at the second and third, but not first resting state scan. Bars ±1 S.E.M. * $p< 0.05$. rMDD – Remitted individuals with a history of recurrent MDD (*n* = 25). HC – Healthy controls (*n* = 25). VS - Ventral striatum. mPFC - Medial prefrontal cortex.

**Supplementary Figure 3**

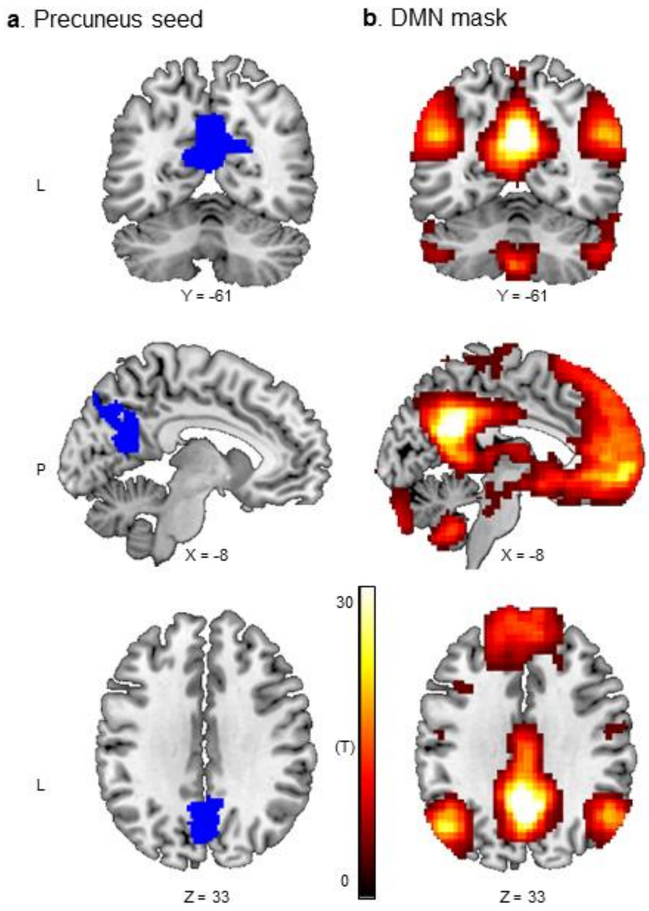


Supplementary Figure 3 - DMN mask. (**a**) Location of the anatomically defined precuneus seed, which was used in a whole brain ANOVA across groups and resting-state time points in order to identify the DMN mask (**b**) at a significance level adjusted such that Type I error was controlled for all voxels in the brain using the false discovery rate (FDR), with $q = 0.05$ in more than 10 contiguous voxels. DMN - Default mode network.

**Supplementary Table 1**

| Group | Acute Phase Descriptive | Acute Phase Captions | Sustained Phase Descriptive | Sustained Phase Captions |
|---|---|---|---|---|
| | | **Reaction time (sec)** | | |
| HC | 11.12 (1.46) | 10.32 (2.51) | 10.49 (2.17) | 10.01 (2.15) |
| rMDD | 10.30 (3.61) | 9.53 (2.23) | 9.33 (3.53) | 9.93 (2.39) |
| | | **Number of trials without a selection** | | |
| HC | 1.72 (1.55) | 1.28 (2.23) | 1.10 (1.32) | 1.24 (2.82) |
| rMDD | 1.27 (1.42) | 0.68 (2.10) | 0.86 (0.92) | 1.00 (3.12) |
| | | **Total number of cursor movements** | | |
| HC | 1.20 (0.36) | 1.34 (0.43) | 1.35 (0.49) | 1.36 (0.50) |
| rMDD | 1.49 (0.57) | 1.29 (0.61) | 1.50 (0.57) | 1.54 (0.76) |

**Supplementary Table 1.** Group averages for three different measures of task-related performance: Length of time needed to make a selection (reaction time, in sec), number of trials without a selection (out of 18), and total number of cursor movements needed to make a selection. For each of these variables, a mixed ANOVA with Group (Controls vs. rMDD) as a between-subject factor, and Condition (Caption vs. Descriptive) and Phase (Acute vs. Sustained) as repeated measures was conducted. No significant effect of Group (all $F$'s$_{(1,49)}$ < 1.85, all $P$'s > 0.18), Condition (all $F$'s$_{11,49)}$ < 0.77, all $P$'s > 0.38), or Group by Condition emerged (all $F$'s$_{(1,49)}$ < 2.83, all $P$'s > 0.10). Taken together, these analyses suggest that task difficulty did not differ across tasks or between groups.

**Supplementary Table 2**

| Group | Comments | Group | Comments |
|---|---|---|---|
| HC | Very positively | rMDD | Made me more positive |
| HC | Made me feel like I have a good sense of humor and can read into things pretty well | rMDD | Positively |
| HC | Positive | rMDD | Positive effect |
| HC | Positively | rMDD | It helped me to motivate |
| HC | It felt good | rMDD | It made me feel good |
| HC | Was nice | rMDD | Enhanced my performance |
| HC | Positively | rMDD | It was nice |
| HC | Motivated me to continue performing well | rMDD | Made me feel proud |
| HC | It made me feel pretty confident | rMDD | It was very uplifting and encouraging |
| HC | It made me feel proud about myself | rMDD | Calmed me |
| HC | Showed that I was doing the task correctly | rMDD | Felt fine hearing it |
| HC | In a positive way | rMDD | It made me feel good |
| HC | I felt good about it and surprised | rMDD | Positively |
| HC | It made me smile | rMDD | More positive |
| HC | Excited and happy | rMDD | Reinforcement |
| HC | Boosted my emotional state | rMDD | Without positive feedback I would have second guessed my decision |
| HC | Unexpected and good | rMDD | It kept me in a positive mind |
| HC | It made me more focused | rMDD | Made me enjoy this task |

**Supplementary Table 2.** Participants' responses after study completion to an open-ended questions whether they perceived the positive feedback as reliable, and if so how did it make them feel. rMDD - Remitted individuals with a history of recurrent MDD; HC - healthy controls.
